# Seychelles warblers with silver spoons: Juvenile body mass is a lifelong predictor of annual survival, but not annual reproduction or senescence

**DOI:** 10.1002/ece3.9049

**Published:** 2022-07-03

**Authors:** Thomas J. Brown, Hannah L. Dugdale, Martijn Hammers, Jan Komdeur, David S. Richardson

**Affiliations:** ^1^ School of Biological Sciences University of East Anglia Norwich UK; ^2^ Groningen Institute for Evolutionary Life Sciences University of Groningen Groningen The Netherlands; ^3^ Nature Seychelles Victoria, Mahé Seychelles

**Keywords:** aging, avian, body mass, juvenile condition, senescence, silver‐spoon, wild population

## Abstract

The environment experienced during development, and its impact on intrinsic condition, can have lasting outcomes for individual phenotypes and could contribute to variation in adult senescence trajectories. However, the nature of this relationship in wild populations remains uncertain, owing to the difficulties in summarizing natal conditions and in long‐term monitoring of individuals from free‐roaming long‐lived species. Utilizing a closely monitored, closed population of Seychelles warblers (*Acrocephalus sechellensis*), we determine whether juvenile body mass is associated with natal socioenvironmental factors, specific genetic traits linked to fitness in this system, survival to adulthood, and senescence‐related traits. Juveniles born in seasons with higher food availability and into smaller natal groups (i.e., fewer competitors) were heavier. In contrast, there were no associations between juvenile body mass and genetic traits. Furthermore, size‐corrected mass—but not separate measures of natal food availability, group size, or genetic traits—was positively associated with survival to adulthood, suggesting juvenile body mass is indicative of natal condition. Heavier juveniles had greater body mass and had higher rates of annual survival as adults, independent of age. In contrast, there was no association between juvenile mass and adult telomere length attrition (a measure of somatic stress) nor annual reproduction. These results indicate that juvenile body mass, while not associated with senescence trajectories, can influence the likelihood of surviving to old age, potentially due to silver‐spoon effects. This study shows that measures of intrinsic condition in juveniles can provide important insights into the long‐term fitness of individuals in wild populations.

## Graphical abstract

Intrinsic and extrinsic factors in early‐life can have a large impact on later‐life fitness and ageing, but the direction and reason for this effect in wild populations is uncertain. We tested whether juvenile body mass influences senescence in a wild population of Seychelles warblers. Heavier juveniles had higher rates of annual survival in adulthood, independently of age and annual reproduction ‐ indicating a silver‐spoon effect of juvenile body mass on adult fitness, but not on senescence onset/rate.

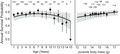

## 1 INTRODUCTION

Senescence—defined as the decline in fitness‐related traits with advancing age—is widespread across the tree of life (Jones et al., 2014). However, longitudinal studies have demonstrated that, even within the same species, individuals can show considerable variation in their onset and rate of senescence in natural environments (Nussey, Froy, Lemaitre, Gaillard, & Austad, 2013; Williams et al., 2006). Identifying drivers of this individual variation is vital for understanding the causes and evolution of senescence. Environmental exposure during development—from conception to maturity—is of particular importance in modulating adult phenotypes (Lindström, 1999; Taborsky, 2006; Vaiserman, Koliada, & Lushchak, 2018). However, there remains uncertainty regarding how differences in developmental environment and/or condition affect senescence.

Body mass and derived indices (e.g., size‐corrected mass) are commonly used measures of individual condition in ecological studies. Body mass has a strong positive correlation with body fat content—the main component of energy storage—and the structural size of individuals (Hayes & Shonkwiler, 2010; Labocha and Hayes, 2012a; Schulte‐Hostedde, Zinner, Millar, & Hickling, 2005). In juveniles, being heavier, fatter, and larger often reduces vulnerability to predation (at least in non‐flying organisms—see below), food‐shortages, and cold‐weather events, and can provide a competitive advantage over peers (Arendt, 1997). Higher body mass is associated with factors that improve the quality of natal socioenvironmental conditions, such as higher food abundance, provisioning effort, and lower densities of competitors (e.g., Bebbington et al., 2017a,b; Mumme, Bowman, Pruett, & Fitzpatrick, 2015). Furthermore, high juvenile body mass is often linked to beneficial genetic traits, such as heterozygosity (e.g., Coltman, Bowen, & Wright, 1998) and immune gene diversity (Arct et al., 2017; Coltman et al., 1998; Kim, Fargallo, Vergara, & Martínez‐Padilla, 2013; Lukasch et al., 2017). As a result, it is common for juvenile body mass to be positively associated (directly and/or indirectly) with survival to adulthood, at least in birds and mammals (Ronget et al., 2018; Tinbergen & Boerlijst, 1990).

There has been extensive debate concerning the quantification of condition (Frauendorf et al., 2021; Green, 2001; Labocha & Hayes, 2012; Labocha, Schutz, & Hayes, 2014; Speakman, 2001; Stevenson & Woods, 2006; Wilder, Raubenheimer, & Simpson, 2016), and it is agreed that simple positive correlations between natal environment, body mass, condition, and fitness cannot be assumed without validation. For example, individuals may retain more fat (resulting in greater body mass) in response to less plentiful and/or predictable food supplies (Cuthill et al., 2000; Marshall et al., 2017). Furthermore, excessive fat deposition (i.e., high body mass relative to size) can reduce an individual's ability to evade predators, especially for flying organisms (Covas, Brown, Anderson, & Brown, 2002; Gosler, Greenwood, & Perrins, 1995). Therefore, the “optimal” body mass (i.e., that which corresponds to highest condition) may be less than the maximum achievable body mass (Barnett, Suzuki, Sakaluk, & Thompson, 2015).

Juvenile body mass, and the factors associated with it, can have lasting effects on adult condition and fitness (e.g., Bowers et al., 2014). Factors that restrict the normal fat deposition and growth rates of juveniles, such as adverse natal environments, generally have negative fitness outcomes (Hsu, Dijkstra, & Groothuis, 2017; Metcalfe & Monaghan, 2001; Seress, Sándor, Evans, & Liker, 2020). For example, in captive zebra finches (*Taeniopygia guttata*) juveniles reared on poor‐quality diets have lower body mass, but also lower reproductive success (Blount et al., 2006; Haywood & Perrins, 1992) and shorter adult life spans (Birkhead, Fletcher, & Pellatt, 1999). Conversely, better natal socioenvironmental conditions, which tend to contribute positively to juvenile mass, can benefit multiple fitness‐related traits in adulthood (Cooper & Kruuk, 2018; Lindström, 1999; Pettorelli et al., 2002); a phenomenon referred to as the “silver‐spoon effect” (Monaghan, 2008). Furthermore, since mass is often positively associated with beneficial genetic and immunological traits, juvenile body mass can be indicative of long‐term individual quality (Gaillard, Festa‐Bianchet, Delorme, & Jorgenson, 2000; Gleeson, Blows, & Owens, 2005; Sakaluk et al., 2015).

Associations between juvenile body mass and adult fitness mean that juvenile body mass could also be informative of adult fitness late in life. For example, silver‐spoon effects can result in delayed (or reduced) senescence when the fitness benefits persist into late life (Cooper & Kruuk, 2018; Nussey, Kruuk, Morris, & Clutton‐Brock, 2007; Pigeon, Festa‐Bianchet, & Pelletier, 2017). Furthermore, where body mass is linked to individual quality, higher mortality rates of lower quality adults (i.e., selective disappearance) would result in a positive relationship between juvenile body mass and longevity (Bowers et al., 2014; Nussey, Coulson, Festa‐Bianchet, & Gaillard, 2008). Alternatively, juvenile body mass can be negatively associated with longevity and late‐life fitness due to trade‐offs with early‐life fitness (e.g., Hunt et al., 2004; Spagopoulou et al., 2020). A greater allocation of resources into growth and reproduction during early adult life can be costly to somatic maintenance and, consequently, later‐life fitness (Hammers, Richardson, Burke, & Komdeur, 2013; Kirkwood, 1977; Lemaitre et al., 2014). Hence, juveniles that are heavier/larger, or that have grown at faster rates can have reduced fitness in late life (Kraus, Pavard, & Promislow, 2013; Metcalfe & Monaghan, 2003; Miller, Harper, Galecki, & Burke, 2002). Likewise, in contrast to silver‐spoon effects, harsh or restrictive natal environments can generate more resilient adult phenotypes (e.g., “thrifty phenotype hypothesis”; Hales & Barker, 2001) or remove individuals with less‐resilient phenotypes at younger ages (selection hypothesis; Nol & Smith, 1987), resulting in individuals that are more resistant to fitness declines in late life (Marshall et al., 2017).

One proposed mechanism by which both natal adversity and growth investments can accelerate senescence is telomere attrition. Telomeres are repetitive nucleotide sequences at the ends of chromosomes, which protect the functional integrity of the genome. In many taxa, telomeres shorten progressively with age (Barrett, Burke, Hammers, Komdeur, & Richardson, 2013; Bendix et al., 2014; Stier, Reichert, Criscuolo, & Bize, 2015) and with increased exposure to various stressors (Chatelain, Drobniak, & Szulkin, 2020; Sudyka, 2019; Young, 2018). Furthermore, shorter telomeres and/or faster rates of telomere attrition are frequently associated with increased mortality risk (Barrett et al., 2013; Fairlie et al., 2016; Vera, Bernardes de Jesus, Foronda, Flores, & Blasco, 2012). Hence, telomere length has been advocated as a marker of life‐history costs to somatic maintenance (Wilbourn et al., 2018; Young, 2018). Both natal adversity and greater investments in early‐life growth tend to be associated with accelerated telomere shortening (reviewed in Monaghan & Ozanne, 2018). Therefore, juvenile body mass/growth and linked natal factors can have long‐term somatic costs, and thus contribute to senescence.

Most previous studies concerned with the impact of natal conditions on senescence use proxies of food availability or closely related factors (e.g., population density and weather) to summarize natal environment. A recent meta‐analysis of such studies found that good natal environments are more often associated with slower rates of reproductive (but not survival) senescence in wild populations; suggesting persistent silver‐spoon effects are more prevalent (or detectable) than early‐ versus late‐life fitness trade‐offs (Cooper & Kruuk, 2018). However, natal condition is a multifaceted concept, involving a suite of both extrinsic and intrinsic factors, which is unlikely to be captured by a limited number of broad‐scale environmental measures. As discussed, juvenile body mass in wild populations can reflect multiple aspects of the natal environment, individual quality, and physiological determinants of survival, for example, energy stores. However, surprisingly few studies have assessed whether natural variation in juvenile body mass is associated with long‐term fitness or senescence; perhaps owing to the difficulty of monitoring individuals across their entire life course in most wild populations (but see Lewin, Swanson, Williams, & Holekamp, 2017).

In this study, we determine whether the body mass of juveniles predicts senescence‐related traits in adult Seychelles warblers, *Acrocephalus sechellensis*—a small insectivorous passerine endemic to the Seychelles. The closely monitored, closed population on Cousin Island is uniquely suited for this study; each individual has annual measures of survival and reproduction, and repeated measures of condition starting from juvenile age. Furthermore, this system benefits from extensive environmental, social, genetic, and physiological data from which the relative contributions of factors to juvenile body mass can be determined. Specifically, we explore the relationship between juvenile body mass and survival to adulthood while accounting for differences in natal food availability, group size, and important genetic traits. Second, we determine whether juvenile body mass is predictive of declines in condition (body mass and telomere length) and fitness (annual survival and reproduction) in late life. This study will contribute to our understanding of the role that natal condition plays on individual variation in senescence in wild populations.

## 2 METHODS

### 2.1 Study species and data collection

The Seychelles warbler is a small insectivorous passerine endemic to the Seychelles. The population on Cousin Island (29 ha; 4°20′S, 55°40′E)—containing ca. 320 adult individuals at any given point (Brouwer et al., 2009)—has been extensively monitored since 1985 (Hammers et al., 2015; Komdeur, 1992; Sparks et al., 2020a). Since 1997, nearly all individuals (>96%) have been ringed with a unique combination of a British Trust for Ornithology (BTO) metal ring and three color rings for easy identification (Raj Pant et al., 2020a; Richardson, Jury, Blaakmeer, Komdeur, & Burke, 2001). Individuals are usually first caught as nestlings, or as dependent juveniles (<5 months old) in their natal territory using mist nets (see Kingma, Komdeur, Hammers, & Richardson, 2016 for details). Juveniles are aged as fledglings (1–3 months), old fledglings (3–5 months), or sub‐adults (5–8 months) based on behavior and eye color (Komdeur, 1992). In addition to capturing unringed juveniles, as much of the ringed adult population as possible (normally ca. 35%) is re‐captured and sampled during the major breeding season (June–September) each year.

The population is structured into ca. 115 clearly defined territories (Kingma et al., 2016), each containing a socially monogamous dominant pair. However, the Seychelles warbler is a facultative cooperative breeder; thus, ca. 50% of territories contain 1–5 sexually mature subordinates (which are usually, but not always, past offspring of the dominant pair), of which ca. 20% of males and ca. 42% of females engage in helping behavior and cobreeding (Hammers et al., 2019; Richardson, Komdeur and Burke, 2003b). Each year, during the major breeding season, each territory is visited at least every 2 weeks to identify all individuals present and determine their status through behavioral observations (Richardson, Burke and Komdeur, 2003a). During visits, the dominant female is followed for ≥15 min to assess breeding activity (Richardson, Burke, & Komdeur, 2007). The majority of breeding activity (94% of territories) occurs from June to August, but a minor breeding season also occurs from January to March (Komdeur & Daan, 2005). Most breeding attempts involve one‐egg clutches (Komdeur, 1994a) but clutches of two or three eggs occur (Richardson et al., 2001). The extensive duration of parental care (ca. 3 months post‐fledging), relative to the length of breeding seasons, limits the opportunity for multiple successful breeding attempts (Komdeur, 1996b). As a result, the majority of successful territories produce just one clutch per breeding season.

In both males and females, annual reproductive success follows a bell‐shaped relationship with age; increasing until 6–8 years‐of‐age before declining in older age (Hammers, Richardson, Burke, & Komdeur, 2012; Raj Pant et al., 2020a). The resighting probability of adults during the major breeding season is close to one (0.98 ± 0.01 SE; Brouwer et al., 2010) and dispersal from the island is virtually absent (Komdeur et al., 2004). Therefore, individuals that are not observed during the major breeding season can be confidently assumed dead. First‐year survival is 0.61 ± 0.09 SE, increasing to a relatively stable 0.84 ± 0.04 SE annual survival in adults (Brouwer, Richardson, Eikenaar, & Komdeur, 2006), before declining from ca. 7 years‐of‐age, that is, the onset of survival senescence (Hammers et al., 2013, 2015). In elderly females, reproductive success is also lower in the last year of life (“Terminal year effect”), suggesting that elderly females are in poorer physiological condition prior to death (Hammers et al., 2012).

During capture events, body mass is measured using either a Pesola or electronic scale (±0.1 g) and structural size is measured using sliding calipers (±0.1 mm) as the length of the right tarsus. Approximately 25 μl of blood is taken from the brachial vein and stored in 100% ethanol (Richardson et al., 2001). DNA extracted from blood samples (following Richardson et al., 2001) is used to confirm sex, using up to three sexing markers, and assign parentage using MasterBayes 2.52 (Hadfield et al., 2006) based on genotypes derived from 30 microsatellite loci (for details see Sparks et al., 2020b). Genetic parentage was used to calculate reproductive success of adults, since estimates based on parental behavior (i.e., incubating, provisioning) are confounded by high rates of extra‐pair paternity (ca. 41%; Raj Pant, Komdeur, Burke, Dugdale, & Richardson, 2019) and co‐breeding among dominant and subordinate helper females (Bebbington et al., 2018; Richardson et al., 2001). Genetic parentage is an underestimation of reproductive success since we excluded offspring for which parents could not be assigned (c. 15% of offspring) and some offspring are likely to have died before being sampled (Edwards, Burke, & Dugdale, 2017). Relative Telomere Length (RTL; the concentration of amplified telomeric DNA relative to that amplified at GAPDH – a single copy gene) has also been measured as part of a previous study (for details see Spurgin et al., 2018). As with many other species, Seychelles warbler telomere length declines with age and with increased exposure to various stressors (Barrett et al., 2013; Spurgin et al., 2018).

In most years of the study (except 2000–2002 and 2005), the availability of food was calculated per territory per field season (following Komdeur, 1992). Briefly, the number of arthropods (on the undersides of leaves) was multiplied by the percentage cover of broad‐leaf vegetation within territories. From this data, we calculated two metrics of food availability, (1) island food availability—mean arthropod abundance across territories during the juvenile's natal field season, and (2) local food availability—arthropod abundance within the juvenile's natal territory. Local food availability was corrected for seasonal differences in overall food availability by subtracting island food availability; thus, positive/negative values of local food availability represent above/below‐average arthropod abundance in the natal territory, respectively, relative to seasonal arthropod abundance. For each natal territory, we also calculated group size (i.e., the number of resident individuals) as a measure of local density, reflecting the intensity of local competition (Brouwer et al., 2006).

### 2.2 Statistical analysis

All analyses were performed in RStudio (version 1.2.5033 and R version 4.0.3, Rstudio Team, 2020). We selected all individuals with biometric data at post‐fledging juvenile age (3 weeks to 5 months after hatching). This is just after the developmental period when skeletal growth is complete (Komdeur, 1991), when juveniles are still dependent on the adults from the natal territory, and before sexual maturity (ca. 8 months; Komdeur, 1997). The Seychelles warbler is sexually dimorphic, with males being larger than females (Safford, 2013). Body mass, as well as being higher in males than females, is also positively correlated with structural size (tarsus length; Kingma et al., 2016).

### 2.3 Juvenile body mass and survival

**FIGURE 1:**
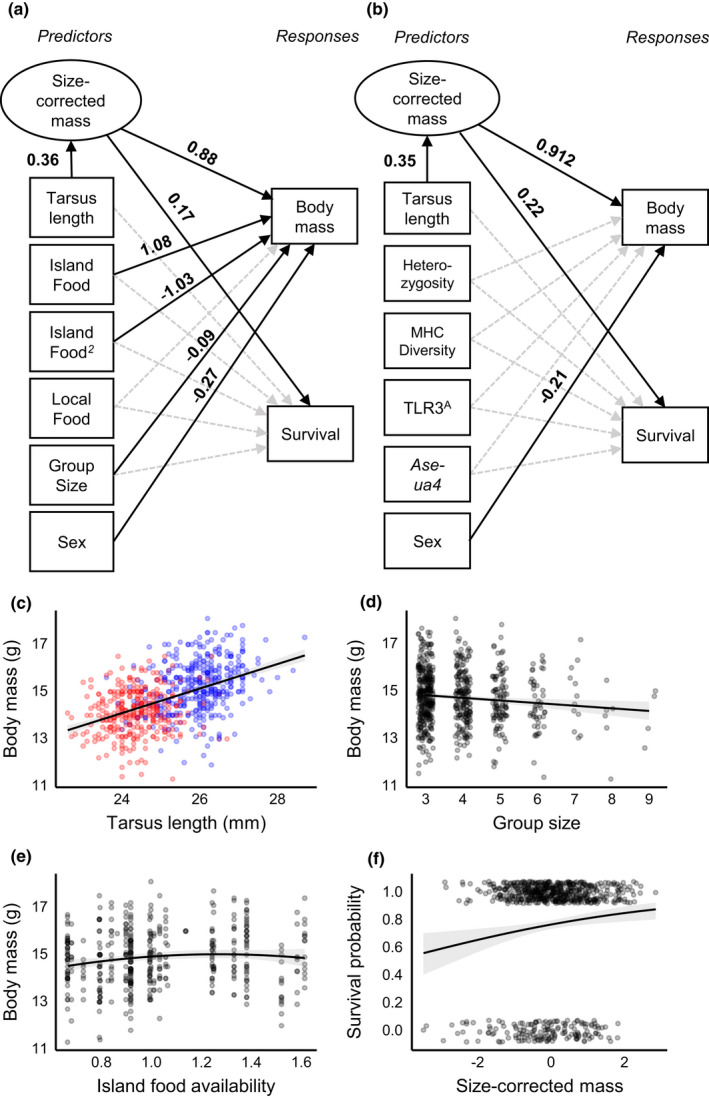
SEM models of natal intrinsic and extrinsic condition in juvenile Seychelles warblers. (a) and (b) are graphical representations of paths between measured variables (in boxes) and the latent variable size‐corrected mass (in oval). Black arrows symbolize significant (*p* < 0.05) paths, with values of standardized coefficients shown next to each line. Standardized coefficients are estimates expressed in equivalent units so that the strengths of different paths can be compared. Grey broken arrows symbolize non‐significant paths. Model (a) incorporates socio‐environmental factors (*n* = 428), while model (b) incorporates genetic traits previously associated with Seychelles warbler survival and fitness (*n* = 233). Plots (c), (d), (e) and (f) visualize significant relationships of interest as estimated by the SEM (a). The fit lines are regressions ‐ linear in (c) and (d), quadratic in (e) and binomial in (f) ‐ between y‐ and x‐axises, with 95% confidence limits. Points are raw data, which in (c) are red for males and blue for females. Y‐axis in (f) is the probability of surviving to > 1 year of age (Y/N)

To conceptualize juvenile condition in terms of body mass and natal socioenvironmental factors, we used a structural equation model (SEM) or path analysis (package lavaan v0.6‐10; Rosseel, 2012), as advocated by Frauendorf et al., 2021. Specifically, we determined the relative contributions of body mass, food availability (island and local), and group size to juvenile survival, and (for food availability and group size) whether contributions to survival occurred directly and/or indirectly via associations with body mass (see Figure 1a). These factors were included due to previously reported associations with Seychelles warbler survival (Brouwer et al., 2006; Hammers et al., 2013). The benefit of a SEM approach over multiple regression models is that juvenile body mass can be a response (i.e., regressed on other variables) and predictor of juvenile survival simultaneously (Figure 1). This removes the need to extract residuals from one regression model to be repurposed as a predictor in a second regression model—a practice which has received extensive criticism (Freckleton, 2002; Green, 2001). For instance, since body mass is often positively correlated with structural size, past studies have regularly used residuals from body mass regressed on size measures as a condition index, with the expectation that this “size‐corrected” mass better reflects an individual's energy store, that is, fat and protein content (Labocha and Hayes, 2012a; Schulte‐Hostedde et al., 2005). In contrast, within our SEM‐model, we use a latent variable—a theoretical construct for which we have no direct measure (Grace, Anderson, Olff, & Scheiner, 2010)—termed “size‐corrected mass” to estimate the relationship between juvenile body mass and survival while accounting for the effect of size (tarsus length) on mass (see Frauendorf et al., 2021 for detail). We also included a squared function for island food availability as both extreme low and high values (indicating drought and high rainfall, respectively) during the natal season are expected to be detrimental to juvenile body mass and survival (Brouwer et al., 2006). We used the maximum likelihood estimation WLSMV to account for the binomial error structure of juvenile survival, defined as whether or not the individual survived to >1 year‐of‐age. So that the strengths of different paths could be compared, we calculated standardized coefficients which are estimates expressed in equivalent units (Figure 1).

As well as investigating the effects of natal socioenvironmental factors, we were interested in whether juvenile body mass, survival, or the body mass–survival relationship were influenced by specific genetic factors. For a smaller subset of individuals in our dataset (233 of 428), information on overall and immunological genetic diversity was available, specifically; heterozygosity, MHC diversity, and the presence of TLR3^A^ and MHC *Ase‐ua4* alleles (yes/no). These traits have been associated with juvenile survival and individual fitness in earlier studies on this species (Brouwer et al., 2010; Davies et al., 2021; Richardson, Komdeur, & Burke, 2004). Using a similar approach as described above, these factors were incorporated into a SEM‐model with juvenile body mass and survival as response variables (Figure 1b). We opted to build a separate SEM‐model (i.e., not including socioenvironmental factors from the previous model) for genetic traits due to a lack of complete data (*n* = 118), and thus concerns of over‐parameterization.

### 2.4 Juvenile body mass, adult body mass, and telomere length

The following analyses on juveniles that survived to adulthood involve testing specific interactions between predictors and the incorporation of random effects (e.g., to account for repeated measures within‐individuals), neither of which are easily accommodated by SEM packages that form latent variables (Frauendorf et al., 2021). Hence, the following analyses were conducted using linear and generalized linear mixed models (package lme4 v1.1‐25; Bates, Mächler, Bolker, & Walker, 2015). We also use juvenile body mass rather than size‐corrected mass as our marker of juvenile condition due to the aforementioned criticisms regarding the use of residuals as predictors (Freckleton, 2002; Green, 2001). Nonetheless, tarsus length was included as a control (i.e., predictor) in all analyses.

First, we determined whether juvenile body mass was associated with two measures of adult condition—body mass and RTL—and or their age‐dependent declines, that is, physiological senescence. Both traits were fitted as responses in two Linear Mixed Models (LMM). Juvenile body mass, adult tarsus length, and sex were included as main effects. In the RTL model, we also included technician identity as a two‐level factor to account for personnel‐related differences in RTL (Sparks et al., 2020b). We used within‐subject centering (van de Pol & Wright, 2009) to separate the role of between‐ versus within‐individual variation with age, that is, cross‐sectional from longitudinal effects. In this way, the individual's age (at measurement of body mass/telomere length) was split into two predictors, (i) mean age across all sampling events for a given individual (mean age), and (ii) within‐individual deviation from mean age (∆age). An interaction term between juvenile body mass and ∆age tested whether juvenile body mass alters the within‐individual slope of adult body mass/telomere length. Since individuals often had multiple measures of adult body mass and telomere length, individual identity was included as a random effect. In the body mass model, observer identity was also included as a random effect to control for possible observer bias in measurements. In the telomere length model, PCR plate identity was included as a random effect to control for possible inter‐plate variation in telomere length (Sparks et al., 2020b).

### 2.5 Juvenile body mass, annual survival, and reproduction

We tested whether juvenile body mass was associated with two fitness components shown to senesce in later adult life in the Seychelles warbler; annual survival and annual reproduction (Hammers et al., 2012, 2013, 2015; Raj Pant et al., 2020). For this analysis, we excluded individuals that had not died by the end of the study period (2019). Furthermore, we excluded the first year of the individual's life, since first‐year survival was covered in our survival to adulthood analysis (see above) and individuals rarely reproduce before 1 year‐of‐age (Komdeur, 1991, 1992). Annual survival was defined as whether or not the individual died before the subsequent main breeding season. Annual reproduction indicated whether the individual produced at least one independent offspring (i.e., surviving to at least 5 months of age) during that year. These fitness traits were fitted as binomial responses (yes vs. no) with a log link function in Generalized Linear Mixed Models (GLMMs). Juvenile body mass was entered as a main effect and as an interaction term with age. A significant main effect would indicate that juvenile body mass influences the fitness component overall, independently of age, while a significant interaction would indicate that juvenile body mass modifies the age‐dependent change in the fitness component. Age (at the end of the main breeding season) was included as a linear and squared term (Hammers et al., 2012; Raj Pant et al., 2020a). To confirm the presence of late‐life declines in survival and reproduction, we repeated analyses including only data above the age of onset of declines; determined visually from non‐standardized squared functions of age (Figures 3a and 4a). Sex and tarsus length were included as additional predictors. Since individuals had multiple measures of fitness, individual identity was included as a random factor. Year was also included as a random factor to control for annual differences in fitness (Brouwer et al., 2006).

In the annual reproduction model, additional predictors were included due to their previously reported associations with annual reproduction and fledging success in this system. An interaction term between sex and age was included due to sex‐specific differences in the onset of reproductive senescence (Hammers et al., 2012; Raj Pant et al., 2020b). Since juvenile body mass is also co‐dependent on sex (see Results), we performed separate male‐only and female‐only models to test whether age‐dependent annual reproduction is influenced by juvenile body mass without the need for overly complex three‐way interactions (i.e., involving sex, juvenile body mass and both linear and squared age terms). Island‐ and local‐food availability were included (for years when these were measured) due to positive associations with fledging success (Hammers et al., 2012). Whether or not the year in question was the last year of an individual's life (terminal year, yes/no) was included, since fledging success was found to be lower in the terminal year of old (≥6 years) females (Hammers et al., 2012). We also included age‐at‐death, to quantify the within‐individual effect of age on reproductive success while controlling for selective disappearance (Hammers et al., 2012; van de Pol & Verhulst, 2006).

In all models, non‐significant interaction terms (coefficient *p*‐value <0.05) were removed sequentially (in order of least significance), so that the first‐order effects could be interpreted, and were only reported if of specific interest. All fixed effects remained in final models (regardless of significance) except for squared functions of continuous variables, which were removed when non‐significant (see Whittingham et al., 2006). Parameter estimates and significance of removed effects were determined by re‐entering them into final models. Continuous fixed effects involved in squared effects and interactions were mean centered to reduce collinearity and aid interpretation (Schielzeth, 2010). Where model singularity errors occurred, we applied maximum a posteriori estimation using blme (v1.0‐5; Dorie, 2013). Where model convergence issues occurred, we used the “BOBYQA” nonlinear optimization (Powell, 2009). Model fit was calculated as conditional *R* ^*2*^ using MuMin (v1.43.17; Bartoń, 2019).

## 3 RESULTS

### 3.1 Juvenile body mass and survival

Juvenile body mass was associated with tarsus length and sex, with larger juveniles and males being heavier (Figure 1c). Juvenile body mass was also associated with natal socioenvironmental conditions. Juveniles from smaller natal group sizes were heavier (Figure 1d) and juveniles born in seasons with moderate food availability were heavier compared juveniles born in seasons of extreme low or high food availability (Table S1; Figure 1e). In contrast, there was no relationship between local‐food availability (i.e., the relative quality of the natal territory) and juvenile body mass (Figure 1a; Table S1). Of the 694 juveniles included in our analysis, 532 survived to adulthood (>1 year‐of‐age). Heavier juveniles (after controlling for their size) were more likely to survive to adulthood (Figure 1f). In contrast, there was no direct effect of island/local food availability or group size on survival, despite associations with body mass (Table S1). Complete data for genetic factors (heterozygosity, MHC diversity, TLR3^A^, and MHC *Ase‐ua4* allele presence), previously shown to influence juvenile survival, were available for 240 juveniles. In our model, none of these genetic factors were associated with juvenile body mass or survival, and the observed juvenile survival–mass relationship remained significant while controlling for these genetic factors (Figure 1b; Table S1).

### 3.2 Juvenile body mass, adult body mass, and telomere length

**FIGURE 2:**
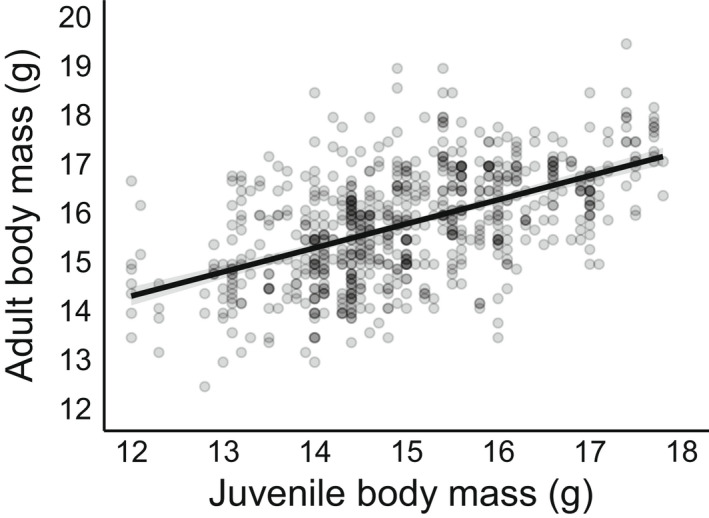
The relationship between juvenile body mass and adult (>1 year of age) body mass in the Seychelles warbler. The fit‐line is a linear regression with 95% confidence limits. Points depict raw data

As expected, adult body mass was higher in males and in increased with tarsus length in both sexes; in a similar manner to that observed with juvenile body mass (Table 1a). While controlling for these factors, adult body mass was positively correlated with juvenile body mass (Table 1a; Figure 2). This indicated that relatively heavier or lighter juveniles tended to remain relatively heavier or lighter, respectively, as adults. Adult body mass increased with age between individuals (i.e., cross‐sectional) and not within‐individuals (longitudinal). These slopes significantly differed (*t* = 2.133, *p* = 0.033), indicating that the between‐individual rate of increase was greater than the within‐individual lack of change (Table 1a). Juvenile body mass did not influence the within‐individual slope of adult body mass (Table 1a; ∆ age × Juvenile body mass).

**TABLE 1:** Linear mixed effects models explaining variation in (a) adult body mass, and (b) relative telomere length (RTL) in the Seychelles warbler

Predictor	Estimate	SE	*t*	*P*
(a) Adult body mass; conditional *R* ^2^ = 0.602				
(Intercept)	8.473	1.347	6.291	<0.001
**Juvenile body mass**	**0.212**	**0.042**	**5.033**	**<0.001**
**Sex (female)**	**−0.706**	**0.108**	**−6.516**	**<0.001**
**Tarsus length**	**0.292**	**0.052**	**5.615**	**<0.001**
**Mean age**	**0.048**	**0.018**	**2.636**	**0.009**
∆ age	−0.005	0.016	−0.308	0.758
∆ age × Juvenile body mass	0.004	0.014	0.270	0.787
Random	711 observations	Variance		
Bird Identity	313 individuals	0.125		
Observer	41 observers	0.044		
Residual		0.505		
(b) Relative Telomere Length (RTL); conditional *R* ^2^ = 0.176				
(Intercept)	1.057	0.363	2.914	0.004
Juvenile body mass	0.009	0.011	0.835	0.406
Sex (female)	0.002	0.029	0.052	0.958
Tarsus length	−0.005	0.014	−0.356	0.722
∆ age	−0.011	0.006	−1.763	0.079
Mean age	−0.009	0.005	−1.950	0.053
**Technician**	**0.081**	**0.022**	**3.728**	**<0.001**
∆ age × Juvenile body mass	0.006	0.005	1.106	0.270
**Random**	**427 observations**	**Variance**		
Bird Identity	207 individuals	0.001		
qPCR plate	70 PCR plates	0.004		
Residual		0.032		

*Note:* Significant effects are in bold.

Relative Telomere Length tended to decrease both within and between individuals with increasing age (Table 1b). The within‐ and between individual rate of change in RTL did not significantly differ (*t* = −1.770, *P* = 0.078). Neither overall adult RTL (Table 1b) nor the within‐individual decline in RTL (Table 1b; ∆ age × Juvenile body mass) was associated with juvenile body mass. RTL was not associated with sex or tarsus length (Table 1b).

### 3.3 Juvenile body mass, annual survival, and reproduction

**FIGURE 3:**
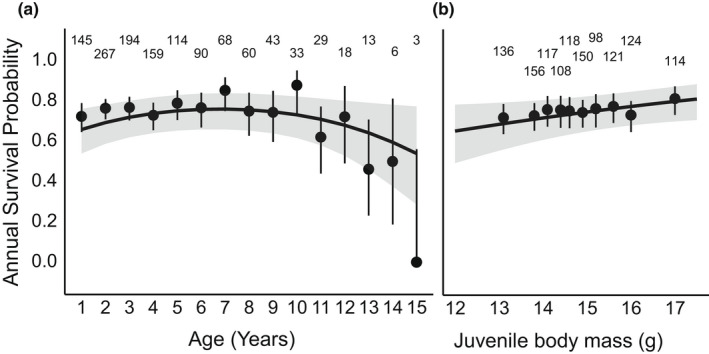
The probability of adult Seychelles warblers surviving to the next year relative to (a) age and (b) juvenile body mass. The fit‐lines are model‐predicted survival curves with 95% confidence limits. Points with error bars are mean survival and binomial 95% confidence intervals of raw data, grouped by (a) age and (b) percentiles of juvenile body mass; note that the x‐axis position of points corresponds to the percentile distribution of juvenile body mass. In text numbers refer to sample sizes per age (c) and percentile of juvenile body mass (b)

Annual survival remained relatively stable at ca. 80% from one to 7 years‐of‐age, beyond which annual survival declined with age (Figure 3a). This decline in annual survival was confirmed by re‐running the analysis with data ≥7 years‐of‐age (β = −0.2523 ± 0.086, *z* = −2.954, *p* = 0.003). Juvenile body mass was positively associated with annual survival, independent of age (Table 2a; Age × Juvenile body mass, Figure 3b). Therefore, heavier juveniles had higher annual survival throughout adult life. Annual survival was not influenced by sex or tarsus length (Table 2a).

**TABLE 2:** General linear mixed effects models explaining variation in (a) annual survival and (b) annual reproduction in adult Seychelles warblers

Predictor	Estimate	SE	*z*	*P*
(a) Annual survival; conditional *R* ^2^ = 0.194				
(Intercept)	−1.666	2.591	−0.643	0.520
**Age**	**0.207**	**0.098**	**2.114**	**0.035**
**Age** ^**2**^	**−0.132**	**0.057**	**−2.297**	**0.022**
**Juvenile body mass**	**0.172**	**0.086**	**1.997**	**0.046**
Sex (female)	0.399	0.211	1.895	0.058
Tarsus length	0.109	0.100	1.084	0.279
Age × Juvenile body mass	0.052	0.094	0.554	0.579
Age^2^ × Juvenile body mass	−0.052	0.058	−0.896	0.371
Random	1242 observations	Variance		
Bird Identity	306 individuals	<0.001		
Year	21 years	0.731		
(b) Annual Reproductive Success; conditional *R* ^*2*^ = 0.287				
(Intercept)	0.415	3.245	0.128	0.898
**Age**	**0.856**	**0.173**	**4.938**	**<0.001**
**Age** ^**2**^	**−0.417**	**0.093**	**−4.502**	**<0.001**
Juvenile body mass	0.129	0.110	1.179	0.239
Sex (female)	−0.120	0.289	−0.414	0.679
Tarsus length	−0.062	0.125	−0.491	0.623
**Terminal year (no)**	**0.718**	**0.210**	**3.414**	**0.001**
Age at death	0.054	0.131	0.414	0.679
**Age × Sex (female)**	**−0.596**	**0.205**	**−2.909**	**0.004**
Age^2^ × Sex (female)	0.071	0.134	0.531	0.595
Males: Age × Juvenile body mass	0.028	0.162	0.175	0.861
Males: Age^2^ × Juvenile body mass	−0.172	0.099	−1.734	0.083
Females: Age × Juvenile body mass	0.325	0.225	1.447	0.148
Females: Age × Juvenile body mass	0.020	0.214	0.095	0.924
Random	1242 observations	Variance		
Bird Identity	306 individuals	0.486		
Year	21 years	0.358		

*Note:* Significant effects are in bold. See Table S2 for sex‐specific analysis of annual reproductive success.

**FIGURE 4:**
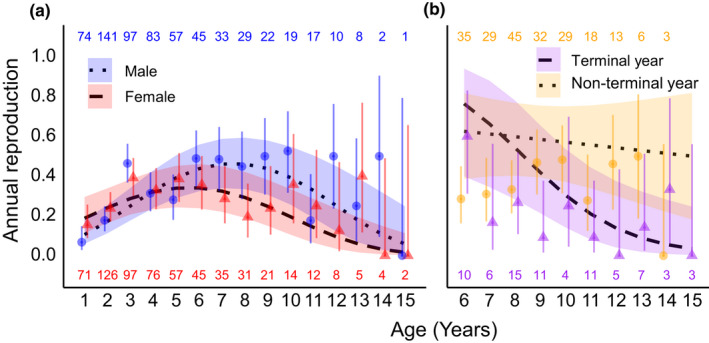
The probability of adult Seychelles warblers producing an independent offspring in a year relative to age and (a) sex and (b) terminal year (yes/no). The fit‐lines are model predicted probability curves with 95% confidence limits. Points with error bars are mean annual reproduction and binomial 95% confidence intervals of raw data, grouped by age per sex (a) and age per terminal year (b). In text numbers refer to the sample sizes per age per grouping variable. Males and females had differing onsets of decline in annual reproduction (a), and the rate of decline was greatest in the terminal year (b)

Annual reproduction was not influenced by island/local food availability; thus these predictors were removed to maximize sample size (*N* = 1034 versus *N* = 1242). Annual reproduction exhibited a humped relationship with age; increasing in early life before peaking and declining from mid‐ to late life (Figure 4a). The age of the peak in annual reproduction (and thus the onset of reproductive senescence) differed between sexes (Figure 4a), with female and male annual reproduction peaking at ca. 6 and 8 years‐of‐age, respectively. Annual reproduction was also lower in the terminal year (Table 2b). Re‐running the analysis on ages from the onset of reproductive senescence (≥6 years for females, ≥8 years in males) confirmed that annual reproduction declined with advanced age, and that the slope of the decline was greatest in the terminal year (Age × Terminal year: β = 0.979 ± 0.396, *z* = 2.471, *p* = 0.013, Figure 4b). Neither annual reproduction nor the age‐dependent change in annual reproduction was influenced by juvenile body mass in males or females (Tables 2b and S2; Age × Juvenile body mass). Annual reproduction was not influenced by tarsus length (Table 2b).

## 4 DISCUSSION

Juvenile body mass was positively associated with better natal socioenvironmental conditions (measured as island food availability and group size) but not with previously identified beneficial genetic traits. Furthermore, juvenile body mass (corrected for size) was, among the socioenvironmental factors and genetic traits tested, the only significant predictor of juvenile survival. For individuals that survived to adulthood, juvenile body mass was positively associated with adult body mass—indicating that individual differences in body mass are maintained from the juvenile period throughout adulthood. More importantly, the survival benefit of being heavier as a juvenile persisted into adult life. Therefore, juveniles that reached adulthood despite being lighter still had poorer survival in a given year compared to adults that were heavier as juveniles. The effect of juvenile body mass on annual survival was constant with age, that is, the age‐dependent decline in survival from 7 years‐of‐age observed in this species did not change with respect to juvenile body mass. Thus, while heavier juveniles are more likely to reach older ages, they still exhibit a similar pattern of survival senescence as those individuals that were lighter as juveniles. There was no observable effect of juvenile body mass on annual reproductive success, nor the maintenance of adult telomere length.

The finding that body mass is positively associated with a juvenile's likelihood of surviving to adulthood in the Seychelles warbler is consistent with findings across birds and mammals (reviewed in Ronget et al., 2018). Our findings suggest high juvenile body mass is indicative of better natal socioenvironmental conditions, which subsequently leads to higher survival. Similarly, previous studies investigating the effects of cooperative breeding in the Seychelles warbler indicate that juveniles receiving nest care from helpers (in addition to parental care) have higher provisioning rates, juvenile body mass, and survival rates than juveniles without helpers (van Boheemen et al., 2019; Hammers et al., 2021; Komdeur, 1994). Similarly, between‐population comparisons of this species suggest that individual body mass on Cousin Island is primarily constrained by food availability and/or population density (Brouwer et al., 2009). Furthermore, experimental studies demonstrate that manipulations of natal/early‐life environments have similar outcomes for juvenile mass/condition and survival in other species (e.g., Grace, Froud, Meillère, & Angelier, 2017; Le Galliard, Ferrière, & Clobert, 2005). Variation in juvenile body mass can also reflect genetic differences which, subsequently, contribute to survival. However, we did not find evidence of this in our study.

Direct benefits of being heavier can also occur where this reflects more abundant energy stores (i.e., fat and protein), since newly independent juveniles lacking experience can be more vulnerable to starvation and exposure (e.g., Jones, Ward, Benson, & Brawn, 2017). Our findings indicate that juvenile body mass is more strongly associated with survival than socioenvironmental factors and known‐genetic traits, which suggests a direct effect of juvenile body mass on survival, perhaps mediated by energy storage. However, body mass (correct for size) can also reflect differences in bodily components other than fat content, such as muscle and organ mass (Labocha and Hayes, 2012a) and genetic constraints. The benefits of high body mass or more abundant energy stores can be traded against increased predation risk, resulting in a body mass–survival relationship that becomes negative with increasing body mass (Adriaensen, Dhondt, Dongen, Lens, & Matthysen, 1998; Blums, Nichols, Hines, Lindberg, & Mednis, 2005). In contrast to these systems, we found that this body mass–survival relationship did not become negative, which was expected given that post‐fledging predation does not occur in this population (Komdeur, 1996a). Therefore, high juvenile body mass, by reflecting better socioenvironments and survival prospects, is indicative of natal condition in this system.

Juvenile body mass was positively correlated with adulthood body mass in the Seychelles warbler, independently of permanent mass constraints such as sex and structural size. This indicates that between‐individual differences in the variable component of juvenile mass (e.g., fat, muscle) are partially maintained across an individual's lifetime. Similar within‐individual consistencies between juvenile and adult mass have been observed in other bird species (Guillemain, Green, Simon, & Gauthier‐Clerc, 2013; Merilä & Svensson, 1997). Previous studies on adult Seychelles warblers have shown that mass is lost during energy‐demanding reproductive behaviors (Bebbington, Kingma, Fairfield, Dugdale, et al., 2017a; Komdeur, 2001; van de Crommenacker, Komdeur, & Richardson, 2011). Therefore, heavier juveniles may be better able to maintain or recover lost energy reserves (i.e., mass) in adult life, perhaps contributing to heavier juveniles also having higher rates of annual survival observed in this study. Conversely, achieving high juvenile body mass at the expense of other physiological components could have negative consequences for adult condition. One potential trade‐off is a greater rate of telomere shortening in early life, resulting in shorter telomere lengths in adulthood (Monaghan & Ozanne, 2018). In many systems, including the Seychelles warbler, short telomeres and/or greater telomere shorting in adulthood also reflects more stressful life histories and reduced survival prospects (Barrett et al., 2013; Hammers et al., 2019; Monaghan & Ozanne, 2018; Wilbourn et al., 2018). However, we found no association between juvenile body mass and adult telomere length, which suggests that the initial benefit of high juvenile body mass does not have long‐term physiological costs, at least when measured with telomere length. This is perhaps not surprising given recent findings in this system demonstrating that telomere dynamics are strongly influenced by the current life‐history pressures faced by the individual (e.g., malarial infections) and thus may poorly reflect differences in natal condition/environment (Brown et al., 2021).

We found that the survival benefits associated with high juvenile body mass were not limited to the first year of life in the Seychelles warbler, with heavier juveniles also having higher annual survival throughout adulthood. This is consistent with our adult body mass analysis, which showed that body mass increased between individuals with age. Silver‐spoon effects of early‐life environment on adult survival have been observed in many wild populations (Alberts, 2019; Cartwright, Nicoll, Jones, Tatayah, & Norris, 2014; Reid, Bignal, Bignal, McCracken, & Monaghan, 2003; Van De Pol et al., 2006). Such effects may occur because juveniles that are heavier and/or reared in better natal conditions have a competitive advantage that leads to them occupying better quality habitat as adults (Verhulst, Perrins and Riddington, 1997; Both, Visser and Verboven, 1999; Van De Pol et al., 2006). In contrast, juveniles that survive to adulthood despite poor‐natal conditions may have required compensatory physiological mechanisms that have delayed survival costs (Briga, Koetsier, Boonekamp, Jimeno, & Verhulst, 2017; Metcalfe & Monaghan, 2001). Juvenile body mass may also reflect differences in intrinsic quality, and thus the longevity, through a combination of environmental (i.e., silver‐spoon effects) and genetic associations (Bowers et al., 2014; Nussey et al., 2008).

The effect of juvenile body mass on annual survival also contributes to lifetime reproductive success, since this is strongly correlated with longevity in the Seychelles warbler (Davies et al., 2021). However, juvenile body mass did not affect the probability of producing offspring in a given year (after controlling for age‐effects) in this species, which is in contrast to studies that have measured the effect of the natal environment on reproductive success (e.g., Douhard et al., 2014; Nussey et al., 2007). In the warbler system, individual breeding attempts are strongly constrained by population density (i.e., limited availability of breeding positions) and seasonal food availability (Komdeur, 1992, 1996c). Additionally, the success of breeding attempts is likely to depend on fine‐scale environmental variation, such as weather, which was not accounted for in this study. Therefore, ecological constraints and confounds may limit the detectable influence of juvenile body mass on annual reproductive success. Furthermore, the strong decline of annual reproduction in the terminal year likely means that poor condition and/or illness in the current year outweighs the effect of past condition (Hammers et al., 2012).

The effect of juvenile body mass on annual survival was constant with age and did not affect the onset or rate of survival senescence. This is consistent with a recent meta‐analysis that found that the quality of early‐life environments was not associated with survival senescence across 18 wild populations (Cooper & Kruuk, 2018). One explanation is that the majority of juveniles that experience poor natal conditions, or are themselves in poor condition, die before reaching senescent age (the age at which a population exhibits reduced survival), while the few individuals that reach old age share traits that mask the effects of natal factors (“selection hypothesis”; Nol & Smith, 1987; Dugdale et al., 2011). Another possibility is that the potential silver‐spoon effect of juvenile body mass is not associated with early‐life investments (e.g., growth, reproductive effort) that have delayed costs for late‐life performance (Hunt et al., 2004; Spagopoulou et al., 2020). For example, (Hammers et al., 2013) identified in this species a trade‐off between early‐life reproductive effort and late‐life survival; individuals that start breeding at earlier ages had an earlier onset of survival senescence. In contrast, our findings suggest that investments in early adult life (in terms of age‐specific annual reproduction) are not associated with juvenile body mass. Therefore, juvenile body mass may fail to generate such resource allocation trade‐offs (i.e., between early‐life reproductive effort and somatic maintenance) that influence senescence patterns.

Our study shows that a juvenile's body mass can be a marker of persistent individual differences in adult condition and performance. This finding reinforces the hypothesis that factors contributing to natal condition can have individual fitness consequences beyond juvenile survival. While juvenile body mass may not predict individual differences in senescence rates, juvenile body mass can be positively associated with longevity, and thus the likelihood of reaching the age at which senescence occurs in wild populations.

## AUTHOR CONTRIBUTIONS

**Thomas James Brown:** Conceptualization (equal); formal analysis (lead); investigation (lead); methodology (lead); writing – original draft (lead). **Hannah Dugdale:** Conceptualization (supporting); data curation (supporting); funding acquisition (equal); methodology (supporting); supervision (supporting); writing – review and editing (supporting). **Martijn Hammers:** Funding acquisition (supporting); methodology (supporting); writing – review and editing (supporting). **Jan Komdeur:** Funding acquisition (equal); writing – review and editing (equal). **David Richardson:** Conceptualization (equal); formal analysis (equal); funding acquisition (equal); investigation (equal); methodology (equal); supervision (lead); writing – original draft (equal); writing – review and editing (equal).

## CONFLICT OF INTERESTS

None declared.

## Supporting information


Appendix S1

## Data Availability

The data that support the findings are openly available in Dryad Digital Repository https://doi.org/10.5061/dryad.ttdz08m1z
