# Supplementary material for: Seychelles warblers with silver spoons: Juvenile body mass is a lifelong predictor of annual survival, but not annual reproduction or senescence
Source: Ecol Evol. 2022 Jul 3;12(7):e9049. doi: 10.1002/ece3.9049 (PMC9251861; doi:10.1002/ece3.9049)
Supplement: Supplementary file 1 — Appendix S1 [file ECE3-12-e9049-s001.docx]

Supplements

| **Table S1**; Regression results from structural equation (SEM) models illustrated in Figure 1. Significant effects are in bold. | | | | |
| --- | --- | --- | --- | --- |
|  |  |  |  |  |
| *Socio-environmental factors*  Model structure:  Size-corrected mass =~ Body mass  Size-corrected mass ~ Tarsus length  Body mass ~ Island food availability + Island food availability^2^ + Local food availability + Sex + Group size  Survival ~ Size-corrected mass + Tarsus length + Local food availability + Island food availability + Island food availability^2^ + Group size | | | | |
|  | Estimate | SE | z-value | P |
| Size-corrected mass ~ |  |  |  |  |
| **Tarsus length** | **0.332** | **0.063** | **5.252** | **<0.001** |
|  |  |  |  |  |
| Body mass ~ |  |  |  |  |
| **Island food availability** | **4.233** | **1.431** | **2.958** | **0.003** |
| **Island food availability^2^** | **-1.807** | **0.627** | **-2.883** | **0.004** |
| Local food availability | 0.001 | 0.155 | 0.006 | 0.995 |
| **Sex** | **-0.56** | **0.127** | **-4.405** | **<0.001** |
| **Group size** | **-0.075** | **0.037** | **-2.064** | **0.039** |
|  |  |  |  |  |
| Survival ~ |  |  |  |  |
| **Size-corrected mass** | **0.193** | **0.069** | **2.784** | **0.005** |
| Tarsus length | 0.206 | 0.111 | 1.862 | 0.063 |
| Local food availability | 0.238 | 0.248 | 0.959 | 0.337 |
| Island food availability | 1.328 | 2.275 | 0.584 | 0.559 |
| Island food availability^2^ | -0.639 | 1.002 | -0.638 | 0.524 |
| Group size | -0.038 | 0.057 | -0.664 | 0.507 |
|  |  |  |  |  |
| *Genetic traits*  Model structure:  Size-corrected mass =~ Body mass  Size-corrected mass ~ Tarsus length  Body mass ~ MHC diversity + Heterozygosity + TLR3A + Aseua4 + Sex  Seen1 ~ Size-corrected mass + Tarsus length + MHC diversity + Heterozygosity + TLR3A + Aseua4 + Sex | | | | |
|  | Estimate | SE | z-value | P |
| Size-corrected mass ~ |  |  |  |  |
| **Tarsus length** | **0.321** | **0.104** | **3.081** | **0.002** |
|  |  |  |  |  |
| Body mass ~ |  |  |  |  |
| MHC diversity | 0.463 | 0.321 | 1.444 | 0.149 |
| Heterozygosity | 0.35 | 0.291 | 1.201 | 0.230 |
| TLR3^A^ | 0.078 | 0.167 | 0.466 | 0.642 |
| Ase-ua4 | 0.248 | 0.159 | 1.563 | 0.118 |
| **Sex** | **-0.417** | **0.207** | **-2.016** | **0.044** |
|  |  |  |  |  |
| Survival ~ |  |  |  |  |
| **Size-corrected mass** | **0.248** | **0.09** | **2.772** | **0.006** |
| Tarsus length | 0.1 | 0.158 | 0.631 | 0.528 |
| MHC diversity | 0.725 | 0.516 | 1.405 | 0.160 |
| Heterozygosity | -0.466 | 0.431 | -1.083 | 0.279 |
| TLR3A | 0.244 | 0.27 | 0.901 | 0.367 |
| Ase-ua4 | 0.084 | 0.226 | 0.37 | 0.711 |
| Sex | 0.125 | 0.312 | 0.402 | 0.688 |
|  |  |  |  |  |

| **Table S2**: General linear mixed effects models explaining variation in annual reproductive success in male (a) and female (b) in adult Seychelles warblers. Significant effects are in bold. | | | | | | |
| --- | --- | --- | --- | --- | --- | --- |
| ***a) Male annual reproductive success;*** conditional *R^2^* = 0.274 | | | | |  |  |
| Predictor | Estimate | | | SE | *z* | *P* |
| (Intercept) | -1.705 | | | 3.936 | -0.433 | 0.665 |
| **Age** | **0.813** | | | **0.185** | **4.405** | **<0.001** |
| **Age^2^** | |  | **-0.402** | **0.094** | **-4.272** | **<0.001** |
| Juvenile body mass | 0.100 | | | 0.131 | 0.759 | 0.448 |
| Tarsus length | 0.017 | | | 0.152 | 0.109 | 0.913 |
| **Terminal year (no)** | **0.932** | | | **0.292** | **3.199** | **0.001** |
| Age at death | 0.100 | | | 0.167 | 0.597 | 0.550 |
| Age × Juvenile body mass | 0.028 | | | 0.162 | 0.175 | 0.861 |
| Age^2^ × Juvenile body mass | -0.172 | | | 0.099 | -1.734 | 0.083 |
| Random | 638 observations | | | Variance |  |  |
| Bird Identity | 158 individuals | | | 0.256 |  |  |
| Year | 21 years | | | 0.299 |  |  |
| ***b) Female annual reproductive success;*** conditional *R^2^* = 0.318 | | | | |  |  |
| Predictor | Estimate | | | SE | *z* | *P* |
| (Intercept) | 3.445 | | | 5.297 | 0.650 | 0.515 |
| Age | 0.341 | | | 0.200 | 1.702 | 0.089 |
| **Age^2^** | |  | **-0.367** | **0.109** | **-3.385** | **0.001** |
| Juvenile body mass | 0.169 | | | 0.192 | 0.879 | 0.379 |
| Tarsus length | -0.185 | | | 0.216 | -0.858 | 0.391 |
| Terminal year (no) | 0.461 | | | 0.316 | 1.462 | 0.144 |
| Age at death | 0.007 | | | 0.212 | 0.035 | 0.972 |
| Age × Juvenile body mass | 0.325 | | | 0.225 | 1.447 | 0.148 |
| Age^2^ × Juvenile body mass | 0.02 | | | 0.214 | 0.095 | 0.924 |
| Random | 604 observations | | | Variance |  |  |
| Bird Identity | 148 individuals | | | 0.856 |  |  |
| Year | 21 years | | | 0.39 |  |  |
